# Effects of Five Lipid Sources on Growth, Hematological Parameters, Immunity and Muscle Quality in Juvenile Largemouth Bass (*Micropterus salmoides*)

**DOI:** 10.3390/ani14050781

**Published:** 2024-03-01

**Authors:** Rui Song, Xinfeng Yao, Futao Jing, Wenxue Yang, Jiaojiao Wu, Hao Zhang, Penghui Zhang, Yuanyuan Xie, Xuewen Pan, Long Zhao, Chenglong Wu

**Affiliations:** 1 National-Local Joint Engineering Laboratory of Aquatic Animal Genetic Breeding and Nutrition (Zhejiang), Huzhou University, 759 East 2nd Road, Huzhou 313000, China; songrui9812@outlook.com (R.S.); xinfengyao666@outlook.com (X.Y.); ywx88899@outlook.com (W.Y.); wujiaojiao5451@outlook.com (J.W.); zhlyyxxf@outlook.com (H.Z.); penghuizhang2021@outlook.com (P.Z.); xieyuanyuan23@outlook.com (Y.X.); a2665469958@outlook.com (X.P.); zl2630903719@outlook.com (L.Z.); 2 Shandong Fisheries Development and Resources Conservation Center, 162 Jiefang Road, Jinan 250013, China; sdyyzzyzk@shandong.cn

**Keywords:** *Micropterus salmoides*, lipid sources, hematological parameters, immunity, muscle quality

## Abstract

**Simple Summary:**

Dietary lipid or oil sources play important roles in modulating growth, immunity and muscle quality in animals. The aim of the present study was to investigate the impact of fish oil (FO), soybean oil (SO), rapeseed oil (RO), peanut oil (PO) and lard oil (LO) on the growth, fish body composition, digestive ability, hematological parameters, serum biochemical indices, immune capability, inflammatory responses and muscle quality in juvenile largemouth bass. Compared with PO and LO, FO and RO showed improvements in weight gain, fatty acid profiles, digestive abilities, innate capabilities and muscle qualities (hardness, firmness and chewiness) with higher collagen synthesizing abilities. The results in this study could provide relative references for the application of dietary lipid sources in largemouth bass and other fish species.

**Abstract:**

This study investigated the effects of fish oil (FO), soybean oil (SO), rapeseed oil (RO), peanut oil (PO) and lard oil (LO) on growth, immunity and muscle quality in juvenile largemouth bass. After 8 weeks, the results showed that FO and RO could increase weight gain and serum alkaline phosphatase and apelin values compared with LO (*p* < 0.05). Except lower crude lipid contents, higher amounts of n-3 polyunsaturated fatty acids (15.83% and 14.64%) were present in the dorsal muscle of the FO and RO groups. Meanwhile, FO and RO could heighten mRNA levels of immune defense molecules (lysozyme, hepcidin, and transforming growth factor β1) compared with PO (*p* < 0.05). While SO could increase potential inflammatory risk via rising counts of white blood cells, platelets, neutrophils and monocytes, and mRNA levels of interleukins (IL-1β, IL-8, IL-12 and IL-15), FO and RO could improve hardness, chewiness and springiness through increasing amounts of hydroxyproline, collagen and lysyl oxidase, and mRNA levels of collagen 1α2 and prolyl hydroxylase in the fish dorsal muscle. Moreover, FO and RO could improve firmness through increasing glycogen and glycogen synthase 1 levels when compared with LO (*p* < 0.05). Therefore, these results could provide dietary lipid source references during the feeding process of adult largemouth bass.

## 1. Introduction

As the main resource of physical capacity and essential fatty acids (EFA), lipid is an important nutrient for fish [1]. In order to ensure normal development, growth and health, fish must obtain enough polyunsaturated fatty acids (PUFAs) derived from food or food precursors [2]. Most vegetable and animal oils could be added into animal artificial feed for their own composition and biological characterizations, such as soybean oil (SO) and peanut oil (PO), rich in n-6 polyunsaturated fatty acids (n-6 PUFAs), and rapeseed oil (RO), rich in monounsaturated fatty acids (MUFAs), while fish oil (FO) is rich in n-3 PUFAs and lard oil (LO) is rich in saturated fatty acids (SFAs) [3]. Although FO is one of the most widely supplemented lipid sources in fish feed due to being rich in n-3 PUFAs and better palatability [4], it has been gradually replaced with vegetable and/or animal oils due to its decreasing yield and higher market price in the world, which has become the most urgent and important focus for rapid and sustainable development of aquaculture.

In the past twenty years, huge amounts of research have estimated the impacts of partial and/or complete FO replacement with vegetable or animal oils in different fish species [5,6]. Recently, many studies have found that FO could be replaced with single or mixed oil sources (vegetable oils and animal oils) without affecting the growth index or feed efficiency in cultured fish species, including silver catfish [7], Nile tilapia [5], rainbow trout [8] and hybrid grouper [9]. However, some studies have also found that vegetable or animal oils could significantly reduce growth performances in European seabass [10] and affect the lipid metabolic ability in gilthead sea bream [11] and bullfrog [4]. This may be due to differences in the fatty acid content of the diet, which in turn has an effect on the growth and metabolic capacity of the fish [4]. In addition, lots of experiments also found there was a close relationship between animal metabolic abilities and adequate levels of serum biochemical indices [12,13]. Lower alanine aminotransferase (ALT) and aspartate aminotransferase (AST) activities could reflect hepatic health status [12]. The higher contents of triglycerides (TG), total cholesterol (TC) and low-density lipoprotein cholesterol (LDL-C) and lower contents of high-density lipoprotein cholesterol (HDL-C) are important hallmarks of dyslipidemia [14]. Apelin (APLN), as an adipokine, has the function of regulating lipid metabolism and can alleviate insulin resistance by affecting the level of adiponectin (ADPN) [15]. In addition, some studies have found that agouti-related protein (AGRP), ADPN and APLN can act as appetite regulators to regulate fish feeding, thereby affecting fish growth [12,16]. Although many studies have reported the relationship between the variations in serum biochemical parameters and dietary lipid sources in fish [17,18], little information about the serum hormone variations could be obtained in fish fed with different lipid sources up to now. This study used changes in serum biochemistry and hormones to examine the impact of lipid sources on the health and appetite of largemouth bass. Meanwhile, there was a close relationship between fish immune status and hematological and serum indices including white blood cells (WBC), hemoglobin (HGB), neutrophils (NEU), monocytes (MON), red blood cells (RBC), platelets (PLT), albumin (ALB), lymphocytes (LYM), hemoglobin (HGB), alkaline phosphatase (ALP), etc. [12,13]. Many studies have demonstrated that higher amounts of RBC, WBC, LYM and HGB could reflect better immunity and effectively promote fish health via the hematopoietic system mediated by adequate nutrients [13,19]. However, little information about the hematological variations could be obtained in largemouth bass fed with different dietary lipid sources up to now. In this study, the immune effects of different lipid sources on largemouth bass were explored through hematological changes so as to provide data support for the choice of lipid source for the health of largemouth bass. Furthermore, some studies also found that the replacement of FO with vegetable or animal oils could significantly influence the muscle texture, smell and taste of fish [20,21]. These studies were mainly focused on marine carnivorous fish, and freshwater herbivorous and omnivorous fish, such as large yellow croaker [22], gilthead sea bream [23], Atlantic salmon [24], turbot [6], grass carp [25,26] and Nile tilapia [27]. However, few studies have focused on the muscle quality of freshwater carnivorous fish fed with different lipid sources.

Largemouth bass is the typical carnivorous economic freshwater fish species originating from North America [28]. It has been broadly farmed since its introduction in China in 1983 and has become an important economic fish because of its higher commercial and nutritional value [17]. In the past forty years, it has become one of the most economic freshwater breeding varieties in China. According to China’s governmental statistics, its culture of largemouth bass achieved 0.8 million tons in 2022 in China [29]. Most studies on the feed of largemouth bass have mainly focused on nutritional requirements, metabolic abilities and innate immunity [17,30,31]. Although there are a few studies on the effect of lipid sources replacing FO in largemouth bass [17,18], few reports could be found detailing a comprehensive comparison of the growth performances, hematological and hormone indices, and muscle quality parameters including muscle texture, odor, histomorphometry and collagen-synthesizing indices in largemouth bass. Therefore, our results will provide a useful theoretical reference for largemouth bass fed with a formulation of artificial feed to improve its muscle quality.

## 2. Materials and Methods

### 2.1. Animal Feeding Diets

Five experimental feeding diets (50.3% crude protein, 9.4% crude lipid) were formulated to contain five lipid sources, including fish oil (FO), rapeseed oil (RO), lard oil (LO), soybean oil (SO) and peanut oil (PO). Casein gelatin and defatted fish meal were explored as the main dietary protein sources. Defatted fish meal (DFM) was obtained from Peruvian red fish meal (lipid 10.8%) with ethanol (95%), then dried at 35 °C and finally contained 1.8% lipids in this experiment. Dextrin was used as a carbohydrate source. All of these ingredients were made into fine powder through 60 mm mesh. The small fraction was mixed by a stepwise expansion method to make pellet feed with a diameter of 2.5 mm. The pellet feed was dried in a hot air circulation oven (Changzhou Innovative Drying Equipment Co., Ltd., Changzhou, China) at 35 °C and stored in −20 °C for further experimental analysis. The ingredients, proximate compositions and fatty acid composition of the experimental diets are presented in Table 1 and Table 2, respectively.

### 2.2. Fish and Feeding Trial

These animal experimental protocols were approved by the Ethical Committee of Huzhou University (Huzhou, China) (approval ID: HUZJ-DW-2021-086; approval date: 16 August 2021) based on the national references for the feed and utilization of vertebrate animals. And these procedures were conducted according to descriptions by Yang et al. [12]. Largemouth bass juveniles were purchased from a commercial breeding farm in Huzhou in August 2021. Before the experimental feeding study, fish juveniles were adapted to indoor culture conditions and fed with the commercial feed (Longshenli, Huzhou, China) in 1000 L fiberglass tanks for 7 days. After measuring and recording the body weight, 450 fish (average weight: 9.30 ± 0.05 g) were randomly assigned into fifteen flow-through fiberglass tanks (500 L) with five triplicates. Each fiberglass tank was stocked with 30 largemouth bass. Largemouth bass were fed with our tested diets containing five different dietary lipid sources, respectively. These diets were fed regularly at 08:00 and 17:00 each day. Fish were fed 3% of their body weight per day. Daily diet amounts were adjusted every 1 week according to the fish weight in each fiberglass tank. During the experimental period (8 weeks), the water was continuously aerated, the temperature was maintained at 27 ± 0.5 °C, the light was natural light, the water PH was approximately 7.2 and the dissolved oxygen of aquaculture water was more than 5.6 mg/L.

### 2.3. Sample Collection and Measurement of Growth Indices

At the trial termination, all fish were fasted and remained stable for at least 24 h. All fish samples were firstly anesthetized with tricane methanesulphonate (≥280 mg/L), and weighed and measured on ice for further analysis of survival (SR), weight gain (WG), special growth ratio (SGR), feed conversion ratio (FCR), protein efficiency ratio (PER) and condition factor (CF) according to the methods described by Yang et al. [12]. A total of 15 fish in each tank were used to determine viscerosomatic index (VSI), intraperitoneal fat ratio (IPF) and hepatosomatic index (HSI). Serum samples were firstly accumulated from the caudal vein and then centrifuged (4 °C, 3000× *g*) for at least 10 min. Dorsal muscle samples from 5 fish were dissected from each fiberglass tank for the texture analysis of muscle texture and quality. Liver, intestine and muscle from 15 fish tanks were also treated according to the methods of Yang et al. [12]. A total of 3 fish in each fiberglass tank were used for analysis of whole-fish body composition analysis and stored at −20 °C.

### 2.4. Assessment of Proximate Composition

All crude protein, crude lipid, moisture and ash amounts in these diets, and the composition of whole-fish body and fish dorsal muscle were measured according to the methods described by Yang et al. [12]. Fatty acid profiles of these experimental diets and fish dorsal muscle samples were all weighed exactly to 20 mg and put separately into 2 mL tubes. A total of 1 mL of mixed solution (dichloromethane/methanol = 1:1) was added to the tubes to grind for 3 min, and the tubes were sonicated at low temperature for 15 min and allowed to stand still at −20 °C for 15 min. After centrifuging at 13,000 rpm for ten minutes at 4 °C, these supernatant samples were transferred into an EP tube and blown dry with nitrogen gas. The EP tube was infused with TMPAH, mixed well and heated at 60 °C in the water bath for 30 min. After cooling at room temperature, hexane was added and centrifuged for 5 min with 8000 rpf. Ultimately, these supernatant samples (100 μL) were transferred to a vial in preparation for measuring by using GC-MS (Agilent Technologies Inc., CA, Santa Clara, USA, 8890B-5977B GC/MSD). Detected data were presented as percentage of total fatty acids.

### 2.5. Assessment of Hematological and Serum Biochemical Parameters

Differential cell types and counts in these blood samples were measured with the TEK 8500 VET automatic blood analyzer (Tekang Technology, Nanchang, China). The values of WBC, mean corpuscular Hb (MCH), RBC, mean corpuscular Hb concentration (MCHC), HGB, PLT, MON, LYM, NEU and mean corpuscular volume (MCV) were recorded. In each group, there were 15 replicates for blood sample analyses. Serum contents or activities of HDL-C, LDL-C, GLU, TG, TC, BUN, alkaline phosphatase (ALP), ALT and AST were measured using a C400n automated hematology analyzer (Shenzhen, China). The content of INS, GC, APLN, ADPN and AGRP in the serum was detected with corresponding commercial kits according to instructions provided by Jiancheng Biotech Co. (Nanjing, China). At least 3 replicates were performed in these analyses.

### 2.6. Measurement of Digestive Enzyme Activities

Liver, intestine and muscle samples were all treated according to the methods described by Yang et al. [12]. These supernatant samples were assembled in a new sterile tube and stored at −80 °C for future analysis. Enzyme activities were also evaluated with corresponding α-amylase (AMS), trypsin and lipase (LPS) detecting kits from Jiancheng Biotech Co. (Nanjing, China). At least 3 replicates were performed in these analyses.

### 2.7. Measurement and Analysis of Muscle Quality

#### 2.7.1. Muscle Texture, Odor and Histomorphometry

The dorsal muscle samples were cut into meat pieces (1 cm × 1 cm × 0.8 cm) for texture determination on scene. Stiffness, toughness, compactness, firmness, chewiness and springiness of the muscles were measured using a Ta. XT Plus texture analyzer (SMS, Surrey, UK) with reference to texture profile analysis (TPA). The probe was P/50 and the test speed was 2.00 mm/s. Muscle samples were immersed, embedded, stained (hematoxylin–eosin) and sealed according to the methods and parameters described by Yang et al. [12]. The myofiber diameter was measured and analyzed with K-Viewer 1.0 software (1.0.4) (https://kv.kintoneapp.com/en/user/, accessed on 26 September 2023).

After the dorsal muscle temperature returned to room temperature, the samples were cut into pieces with scissors, and 3 g samples were weighed into a beaker of 100 mL, sealed with double plastic wrap and left at room temperature for 1 h before muscle odor testing. Muscle odors were determined by direct headspace aspiration, with the injection needle inserted directly into the headspace bottle containing the sample and determined with a PEN3 electronic nose (Airsense, Schwerin, Germany). The measurement conditions were as follows: sample collection time was 1 s/group, sensor self-cleaning time was 80 s, sensor zeroing time was 5 s, sample preparation time was 5 s, injection flow rate was 400 mL/min and analytical sampling time was 80 s.

#### 2.7.2. Collagen Synthesis-Related Indexes and Cathepsin Contents

Using liquid nitrogen, the dorsal muscle specimens were homogenized into powder which was resuspended with physiological saline separately. The content or values of cathepsin-b (Cath-B), proline hydroxylase (PHD), cathepsin-l (Cath-L), lysyl oxidase (LOX) and collagen pyridine cross-linking (PYD) in the dorsal muscle were detected with corresponding commercial ELISA kits (Jiangsu Enzyme-linked Biotech Co., Ltd., Yancheng, China). Hydroxyproline (HYP) content was examined with a commercial detecting kit from Jiancheng Biotech Co. (Nanjing, China). And the measured hydroxyproline content was multiplied by the coefficient 8 to obtain the collagen content.

### 2.8. Measurement and Analysis of Function Gene Expression Variations

All RNA samples were taken from the liver and the dorsal muscle of these different fish groups using Invitrogen Trizol reagent (Carlsbad, CA, USA). All cDNA samples were obtained according to the methods supplied by Wu et al. [29] and then stored at −20 °C for further qPCR analysis. Expression variations in immune genes in the liver, antioxidant genes, collagen-related genes and cathepsins in the dorsal muscle versus β-actin (the internal reference gene) were detected using real-time fluorescence qPCR assays on the Biorad CFX96 thermo cycler (Hercules, CA, USA) (Table 3). Relative transcription variations in these genes were evaluated with 2^−ΔΔCT^ method.

### 2.9. Statistical Analysis

Principal component analysis (PCA) is a common algorithm in dimension reduction [32]. It has become the most commonly used feature extraction method. It is a method of data dimensionality reduction by using linear mapping, and at the same time, it can remove the correlation of data to keep the variance information of the original data to the greatest extent. Principal component analysis (PCA) was performed on standardized data of dorsal muscle with dissimilarities and correlations in the analyzed variables between these samples. The PCA diagram was made based on the method of Mu et al. [21].

All experimental data are presented as mean ± SD (standard deviation). Firstly, one-way analysis of variance (ANOVA) with Tukey’s test was applied for analyzing statistical differences among these different groups with SPSS 26.0 (SPSS Inc., Chicago, IL, USA). And then, the follow-up linear and/or quadratic trends were determined using the orthogonal polynomial contrasts. *p* < 0.05 was used to determine statistical significance.

## 3. Results

### 3.1. Growth Performance

The FBW, WG and SGR in the FO, SO and RO groups were markedly higher than in the PO and LO trial groups (*p* < 0.05) (Table 4). Meanwhile, the lowest FBW, WG, SGR and PER accompanied by the highest FCR values were found in the LO trial groups (*p* < 0.05). The highest VSI and HSI values were observed in the RO trial groups (*p* < 0.05), although no marked variations in HSI were obtained in the SO and LO trial groups (*p* > 0.05). And higher IPF values were present in the SO and LO trial groups (*p* < 0.05). The highest CF values were obtained in the FO and LO trial groups in comparison with the SO, RO and PO trial groups (*p* < 0.05). The highest CR values were shown in the RO trial groups (*p* < 0.05), while the lowest CR values were shown in the LO trial groups (*p* < 0.05). However, there were no marked changes in SR values among fish groups treated with these five lipid sources (*p* > 0.05).

### 3.2. Proximate Composition

Higher moisture values in whole-fish bodies were shown in the LO and RO trial groups (*p* < 0.05). In spite of no marked variation in the crude protein levels in whole-fish bodies among the FO, SO, RO and LO trial groups (*p* > 0.05), the lowest crude protein values were shown in the whole-fish body of the PO groups (*p* < 0.05) (Table 5). The highest crude lipid contents in whole bodies were found in the SO experimental groups (*p* < 0.05), while the minimal crude lipid contents were shown in the PO trial groups (*p* < 0.05). Although the maximal ash values were shown in the PO experimental groups, there were no marked variations in the ash values in whole-fish bodies among the FO, SO, RO and LO groups (*p* > 0.05). Additionally, no marked variations were shown among the FO, SO, RO and PO trial groups, although the lowest moisture levels were shown in the fish muscle of the LO trial groups (*p* < 0.05). Similarly, the maximal lipid values were observed in the dorsal muscle of the LO trial groups (*p* < 0.05), although no marked variations were observed among the FO, SO, RO and PO trial groups (*p* > 0.05). The amounts of crude protein and ash in the dorsal muscle were not impacted among these five lipid trial groups (*p* > 0.05).

The percentages of saturated fatty acids (SFAs), C18:1n-9 (oleic acid, OA) and monounsaturated fatty acids (MUFAs) in the dorsal muscle of the LO experimental groups were markedly higher than those in the FO, SO, RO and PO trial groups, while the minimal amounts of SFAs, OA and MUFAs were present in the FO experimental groups (*p* < 0.05) (Table 6). The highest percentage of C18:2n-6 (linoleic acid, LA) and n-6 PUFAs accompanied by the lowest percentage of C20:5n-3 (eicosapentaenoic acid, EPA) was found in the SO trial groups (*p* < 0.05). The amounts of C18:3n-3 (α-linolenic acid, ALA) were markedly higher in the dorsal muscle of the RO experimental groups than those in the dorsal muscle of the FO, SO, PO and LO groups (*p* < 0.05). The maximal percentage of EPA, C20:6n-3 (docosahexaenoic acid, DHA) and n-3 polyunsaturated fatty acids (n-3 PUFAs) was shown in the dorsal muscle of the FO trial groups, while the lowest percentage of n-3 PUFAs was shown in the dorsal muscle of the PO trial groups, in comparison with the FO, SO, RO and LO trial groups (*p* < 0.05).

### 3.3. Hematological and Biochemical Parameters

The highest amounts of WBC, PLT, MCHC, NEU and MON and the lowest amounts of MCV and LYM were all observed in the SO experimental groups (*p* < 0.05) (Table 7). The highest values of RBC, HGB and LYM were present in the blood of the RO trial groups (*p* < 0.05). The highest values of MCH and MCV and the lowest amounts of WBC and MCHC were all present in the LO trial groups (*p* < 0.05). The lowest counts of NEU were shown in the PO experimental groups, compared to the other four lipid sources (*p* < 0.05). The lowest values of RBC, HGB and MCH were found in the FO trial groups in comparison with the other four lipid sources.

Although there were no marked changes in HDL-C values among the five lipid experimental groups (*p* > 0.05), higher LDL-C contents occurred in the RO and LO experimental groups compared with the FO, SO and PO experimental groups (*p* < 0.05) (Table 8). Lower GLU values were shown in the FO and PO experimental groups, while higher GLU values were observed in the SO, RO and LO experimental groups (*p* < 0.05). The lowest TG contents were present in the FO trial groups, while higher TG levels were observed in the SO and LO experimental groups (*p* < 0.05). Similarly, the lowest TC contents were also present in the FO groups, while higher TC levels were observed in the other four lipid experimental groups (*p* < 0.05). The highest BUN values were observed in the LO experimental groups, while the lowest BUN contents were observed in the RO trial groups (*p* < 0.05). And no marked differences were observed in the BUN contents among the FO, SO and RO experimental groups (*p* > 0.05). The lowest contents of ALB and ALP were found in the SO groups, but the highest ALB values were shown in the LO experimental groups and the highest ALP levels were shown in the RO experimental groups (*p* < 0.05). Furthermore, the lowest activities of AST and ALT were shown in the serum of the RO experimental groups, while the highest AST activities were observed in the SO groups and the highest ALT activities were present in the LO experimental groups (*p* < 0.05).

In addition, there were no marked variations in the levels of INS, ADPN and AGRP among these five lipid groups (Table 9). The lowest GC contents were present in the serum of the FO groups in comparison with the SO and RO experimental groups (*p* < 0.05), although there were no marked variations among the FO, PO and LO experimental groups (*p* > 0.05). Values of APLN in the serum of the FO, SO and RO trial groups were markedly higher than those in the serum of the LO and PO experimental groups (*p* < 0.05).

### 3.4. Digestive Enzymes

There were lower AMS activities in the liver of the LO and PO experimental groups in comparison with the other three lipid source groups (*p* < 0.05), although no marked differences were shown in the AMS activities in the liver of the FO, SO and RO groups (Table 10). Similarly, there were lower TRY activities in the liver of the LO and PO groups in comparison with the SO and RO groups (*p* < 0.05), although no marked differences were observed in the TRY activities in the liver of the FO, SO and RO experimental groups (*p* > 0.05). In addition, the lowest and highest LPS activities were shown in the liver of the LO and FO groups, respectively (*p* < 0.05). And there were no statistical variations in LPS activities among the SO, RO and PO experimental groups. In the intestine, higher AMS activities were shown in the SO and PO experimental groups compared to the other three lipid source groups (*p* < 0.05), although there were no marked changes in the AMS activities in the FO, SO and RO experimental groups (*p* > 0.05). The highest TRY activities were shown in the RO groups, while the lowest TRY activities were present in the PO experimental groups (*p* < 0.05). And there were no notable variations in TRY activities between the FO and SO source groups (*p* < 0.05). Furthermore, no statistical variations were observed in LPS activities in the intestines among these five lipid source groups (*p* > 0.05).

### 3.5. Hepatic Immune and Inflammatory Indices

**Figure 1:**
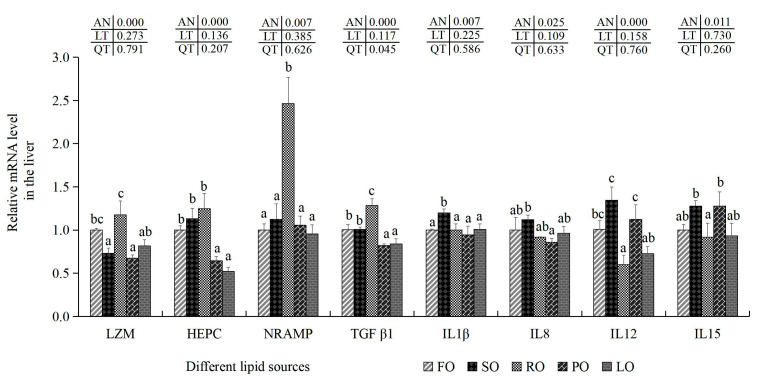
Effects of different dietary lipid sources on related mRNA levels of anti-inflammatory and pro-inflammatory cytokines in the liver of juvenile largemouth bass. Values are means ± SD (*n* = 9). Mean values with different superscripts in the same column are significantly different based on Tukey’s test (*p* < 0.05).

The transcription levels of LZM were markedly decreased to the lowest in the liver of the SO and PO experimental groups in comparison with the FO, SO and RO trial groups (*p* < 0.05) (Figure 1). The maximal transcription amounts of NRAMP, HEPC and LZM were present in the liver of the RO groups (*p* < 0.05). However, transcription levels of IL-1β, IL-8, IL-12 and IL-15 reached the maximal levels in the SO experimental groups (*p* < 0.05). TGF-β1 showed the opposite trend to these pro-inflammatory cytokines (IL-1, IL-8, IL-12 and IL-15) as it was most pronounced in the RO groups (*p* < 0.05).

### 3.6. Muscle Quality

#### 3.6.1. Muscle Texture, Odor and Histomorphometry

**Figure 2:**
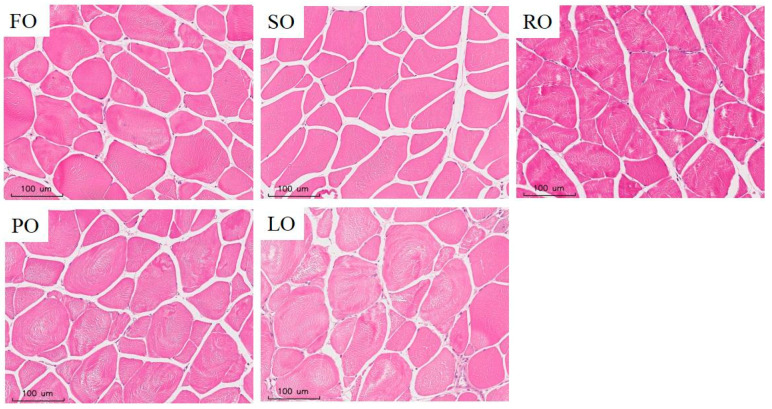
The dorsal muscle sections with HE staining of juvenile largemouth bass fed with diets containing FO, SO, RO, PO and LO (magnification × 200).

The highest hardness, firmness and chewiness were found in the FO groups, while the lowest relative parameters were shown in the LO trial groups (*p* < 0.05) (Table 11). And there were no marked variations in the hardness, firmness and chewiness among the FO, SO and RO experimental groups. There were no marked variations in the springiness values among the FO, SO, RO and PO experimental groups, while the maximal springiness and the minimal springiness were present in the SO and LO experimental groups (*p* < 0.05), respectively. In addition, there were no marked variations in the toughness among these five lipid source groups. The minimal myofiber density was shown in the LO experimental groups, although there were no statistical variations among the FO, SO, RO and PO groups. Higher myofiber diameters were present in the SO, RO, PO and LO groups, while the lowest myofiber diameter was presented in the FO trial groups (*p* < 0.05) (Figure 2). Furthermore, the highest myofiber density compared with the lowest myofiber diameter was found in the FO group.

**Figure 3:**
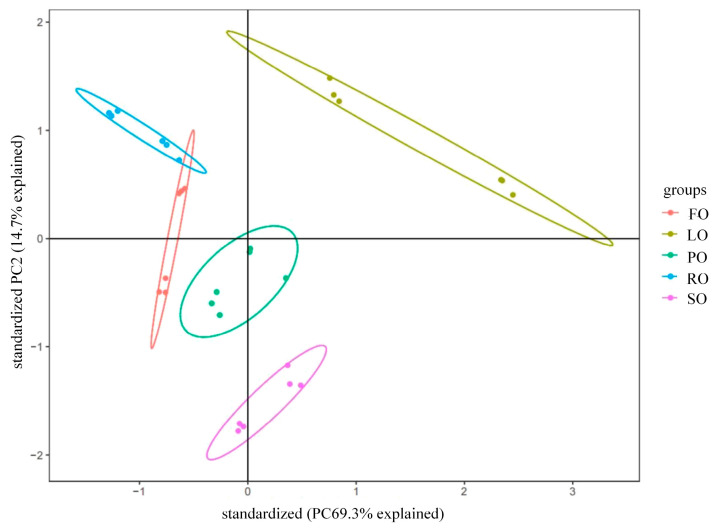
PCA analysis of effect of dietary lipid sources on dorsal muscle odor of largemouth bass.

A PCA plot of muscle odor from various dietary lipid sources is displayed in Figure 3. Principal component 1 (PC1) and principal component 2 (PC2), with a contribution rate of 69.3% for PC1 and 14.7% for PC2, are the two primary component axes displayed in the figure. The total contribution rate was 84%, which reflects the overall odor of the sample. There were significant differences in muscle odor between different dietary lipid sources. The odors of the SO and LO groups were considerably different from those of the FO, RO, and PO groups, although there was no marked differences in muscle odor among the RO, PO and FO groups.

#### 3.6.2. Collagen Synthesis-Related Indexes and Cathepsin Content in the Muscle

**Figure 4:**
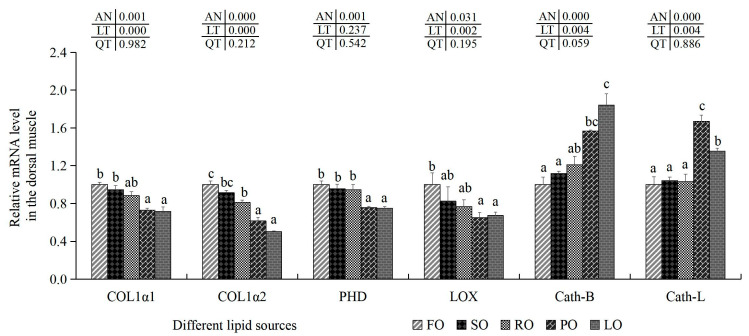
Effects of different dietary lipid sources on related mRNA levels of collagen-related genes and cathepsin in the dorsal muscle of juvenile largemouth bass. Values are means ± SD (*n* = 9). Mean values with different superscripts in the same column are significantly different based on Tukey’s test (*p* < 0.05).

There were higher HYP amounts in the dorsal muscle of the FO and SO groups compared with the PO, RO and LO experimental groups (*p* < 0.05), although no marked variations in HYP were shown among the FO, SO and RO experimental groups (Table 12). The highest contents of collagen and PHD were present in the FO groups, while the lowest contents of collagen and PHD were observed in the LO trial groups (*p* < 0.05). Although the minimal LOX amounts were shown in the PO groups (*p* < 0.05), no marked variations in the LOX contents were shown in the FO and RO experimental groups (*p* > 0.05). Similarly, lower PYD contents were found in the PO groups in comparison with the FO groups (*p* < 0.05), although no statistical variations in the PYD contents in dorsal muscle of the FO, SO, RO and LO experimental groups were found (*p* > 0.05). The minimal Cath-B values were observed in the FO groups, although no marked differences were obtained in the SO experimental groups (*p* > 0.05). Additionally, the maximal values of PHD, LOX and PYD and the minimal Cath-B content were all observed in the FO groups. There were no statistical differences in the Cath-L content among the FO, SO, RO, PO and LO experimental groups (*p* > 0.05). Moreover, the transcription levels of COL1α1, COLl1α2, PHD, Cath-B and Cath-L were notably influenced by dietary lipid sources (*p* < 0.05) (Figure 4). Transcription amounts of COL1α1, COL1α2 and PHD were markedly down-regulated in the dorsal muscle of the PO and LO groups in comparison with the FO trial groups (*p* < 0.05). Although there were no marked variations in the transcription levels of LOX in the muscle of the SO, RO, PO and LO groups, mRNA levels of LOX in the PO and LO groups were significantly decreased compared with the FO groups (*p* < 0.05). However, the transcription levels of Cath-B and Cath-L were both statistically elevated in dorsal muscle of the PO and LO groups compared with the FO and SO trial groups (*p* < 0.05).

#### 3.6.3. Glycogen Synthesis and Antioxidant-Related Indexes in the Muscle

**Figure 5:**
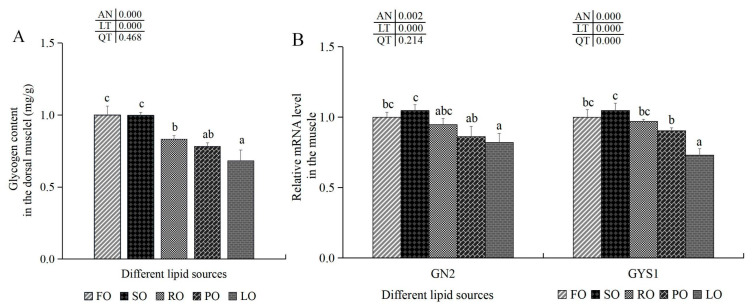
Effects of different dietary lipid sources on glycogen content (**A**) and mRNA expression levels of glycogen synthesis-related genes (**B**) in the dorsal muscle of juvenile largemouth bass. Values are means ± SD (*n* = 9). Mean values with different superscripts in the same column are significantly different based on Tukey’s test (*p* < 0.05).

The highest content of glycogen was shown in the FO group, although there was no statistical variance between the FO and SO experimental groups (*p* > 0.05). Glycogen values in the dorsal muscle of the FO and SO experimental groups were markedly higher than those in the RO, PO and LO experimental groups, and the highest glycogen values were shown in the FO experimental groups (*p* < 0.05) (Figure 4). The transcription levels of GN2 and GYS1 in the dorsal muscle of the PO and LO trial groups were notably lower than those in the FO experimental groups (*p* < 0.05), although no marked variations were found among the FO, SO and RO experimental groups (*p* > 0.05) (Figure 5).

**Figure 6:**
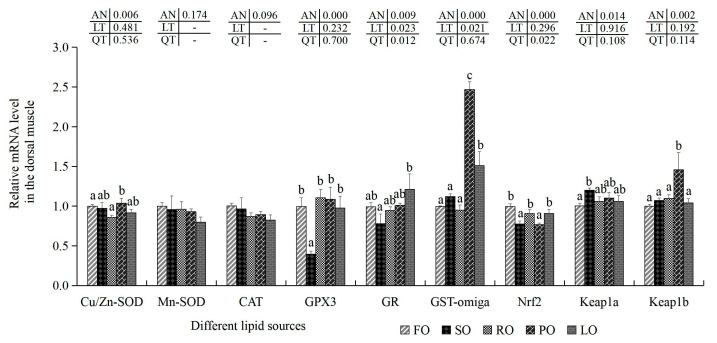
Effects of different dietary lipid sources on related mRNA levels of antioxidant-related genes in the dorsal muscle of juvenile largemouth bass. Values are means ± SD (*n* = 9). Mean values with different superscripts in the same column are significantly different based on Tukey’s test (*p* < 0.05).

There were no notable changes in the transcription amounts of Cu/Zn-SOD in the dorsal muscle of the FO, SO, RO and LO trial groups (*p* > 0.05), while the maximal and minimal amounts of Cu/Zn-SOD were found in the RO and LO trial groups (*p* < 0.05), respectively (Figure 6). There were no marked variations in the transcription amounts of Mn-SOD and CAT among these five lipid source groups (*p* > 0.05). Although there were no statistical variances in the mRNA levels of GPX3 and GR in the FO, RO, PO and LO trial groups, the minimal GPX3 and GR levels were both present in the SO trial groups (*p* < 0.05). Compared with the FO, SO and RO trial groups, the transcription levels of GST-omega were markedly up-regulated in the PO and LO trial groups (*p* < 0.05). In addition, the transcription levels of Nrf2 in the FO, RO and LO trial groups were higher than those in the SO and PO trial groups (*p* < 0.05). Although no marked variations in the transcription levels of Keap1a were shown in the muscle of the FO, RO, PO and LO trial groups, Keap1a transcription levels were significantly heightened in the SO trial groups in comparison with the FO experimental groups (*p* < 0.05). Moreover, the transcription amounts of Keap1b were significantly heightened in the PO trial groups in comparison with the FO, SO, RO and LO experimental groups (*p* < 0.05).

## 4. Discussion

### 4.1. Growth Indices and Proximate Composition

Lipids are a source of essential fatty acids which could promote fish growth [33]. Previous studies have found that SO or RO could effectively replace FO without compromising the growth performance of aquatic animals [34,35]. Similarly, there were no differences in growth performances among the FO, SO and RO groups in our results, which is in line with previous studies on largemouth bass [17,18]. Other studies have proved that ALA can be converted by freshwater fish into LC-PUFA (long-chain polyunsaturated fatty acid), which is the necessary fatty acid for animal normal growth and development [36]. Combined with higher contents of ALA in FO, SO and RO, these three diets could supply enough substrate to synthesize LC-PUFA and then improve the growth of largemouth bass [36,37]. However, the PO and LO groups displayed lower growth performances in our results, which is in line with previously obtained results in European seabass [10] and bullfrog [4]. This may be related to the lower proportion of ALA in PO and LO, which mediates the lack or insufficient amount of dietary ALA, impairing the normal growth and development of fish [36].

Many studies have demonstrated that different fatty acids in lipid sources could impact the body’s approximate compositions [4,38]. In the present study, the maximal lipid content of fish bodies was observed in the SO groups, which is in line with the results obtained for mandarin fish [34]. The increased lipid retention in the SO groups may be due to higher dietary LA content [37,39]. In addition, the maximal lipid content was shown in the dorsal muscle of the LO groups, which is similar to the results obtained for bullfrogs [4]. Previous studies have found that higher dietary SFA contents in LO were more easily used for lipid accumulation or deposition in tissues when compared to dietary MUFAs and PUFAs [38]. Combined with higher IPF and lipid contents in the dorsal muscle, this indicates that higher dietary SFA contents in LO could lead to excessive lipid deposition in the muscle and peripheral adipose tissues in largemouth bass [40]. Similar to previous results in largemouth bass, there were no significant variations in the crude protein and crude ash values in these five lipid source groups, which indicates that these five lipids do not affect protein synthesis and mineral deposition in largemouth bass [17,18,41]. Moreover, dietary fatty acid compositions could affect fatty acid profiles in the dorsal muscle samples in culturing animals [4,42]. Our results have shown that fatty acid profiles in dorsal muscle changed with variations in dietary fatty acid compositions, which is similar to former results obtained with fish species [17,18,41,42,43]. Previous studies have found that the replacement of FO with vegetable oil leads to reduced EPA and DHA contents in the muscle of silver barb [43], beluga sturgeon [44] and glass carp [42]. Considering the variations in DHA and EPA contents in the dorsal muscle of largemouth bass in this study, this indicates that DHA and EPA could be retained in the muscle from diets [45]. And higher retention of DHA over EPA may be due to the selective utilization of EPA over DHA in order to fulfill the needs for membrane structure and performance [20].

### 4.2. Serum Biochemical Parameters

Serum biochemical analyses can serve as dependable indicators for the detection of metabolic abnormalities. Higher serum ALT and AST activities can indicate typical hepatic damage and poor health [46]. Previous studies have found that lower ALT and AST activities were also related to improved liver health [12]. In this experiment, ALT and AST activities were reduced in the serum of the RO trial groups, indicating that dietary RO could improve hepatic health in largemouth bass, which was similar to previous results in hybrid sturgeon [47]. The elevations in TG, TC and LDL-C and decline in HDL-C contents are important hallmarks of dyslipidemia [14]. Consistent with previous results in broiler fish [48], dietary LO could increase serum TG, TC and LDL-C amounts. Previous research has demonstrated that the increase in SFA intake is positively correlated with serum TG and TC contents [49]. Combined with higher SFA contents in an LO diet, it is suggested that LO could heighten serum lipid levels and impact the health of largemouth bass [50]. Meanwhile, reduced BUN levels could indicate better utilization of nitrogen, a balance of amino acids in the diet [51] and the promotion of protein deposition in animals [12]. Previous studies have found that lower BUN levels were observed in dairy cows fed with FO and SO [52]. Similarly, lower BUN levels occurred in the FO, SO and RO trial groups, indicating that FO, SO and RO could increase protein deposition and then promote the growth of largemouth bass. It is well known that hormones are a class of biologically active substances secreted by endocrine glands and play important regulatory roles during metabolic processing in human and animals. Insulin (INS) is the main hormone that reduces serum glucose contents mainly by increasing its uptake of glucose in peripheral tissues [53], while glucagon (GC) increases serum glucose concentrations by promoting the production of glucose by cells from various substrates [54]. This study showed that lower contents of GLU and GC were both found in the FO groups, which is similar with previous results in dog [55]. In general, FO is rich in omega-3 fatty acids, which enhance the sensitive function of ADPN and lower serum glucose content [56]. In addition, vast amounts of research have proved that AGRP, APLN and ADPN could serve as activating factors that enhance fish feeding abilities [16,57,58]. Combined with lower FCR values in the FO and RO groups, it is indicated that FO and RO could promote feed utilization and subsequently improve growth indexes by boosting the levels of feeding-related hormones (AGRP, APLN and ADPN) in largemouth bass [12].

### 4.3. Digestive Enzymes

Digestive enzymes, including AMS, LPS and TRY, always play important roles in digesting nutrients and maintaining the normal growth and development of animals [59]. The content and activity of AMS can determine the ability of fish to digest dietary carbohydrates [60]. In line with results obtained for dairy bulls [61], higher AMS activities were shown in the intestines and liver of the SO groups, which indicates that dietary SO could enhance carbohydrate utilization in largemouth bass [62]. As the dominant enzyme in lipolysis, variations in LPS activities can reflect the animal’s ability to use lipids [63]. In this study, we found that there were lower LPS activities in the intestines and liver tissues in the LO trial groups, which is in line results found with red swamp crayfish [64]. The decreased LPS activities in the LO groups may be due to high levels of saturated fatty acids (C18:0) in LO, which restrict lipid absorption [65]. Normally, TRY, a proteolytic enzyme, can present a close relationship between its higher protease activity and better growth performance in animals [66]. Therefore, higher TRY activities in the liver and intestines of the FO, SO and RO groups indicates better utilization of dietary amino acids, subsequently increasing the growth indexes of largemouth bass [60].

### 4.4. Immune and Inflammatory Indices

A previous study has found that the changes in dietary fatty acid composition could affect the innate immunity and inflammatory responses of fish [67]. In general, LZM, HEPC and NRAMP secreted by these immune cells can improve innate immunity and protect culturing animals from bacterial infection [68]. Numerous studies have discovered that there is a positive association between greater levels of HEPC, LZM and NRAMP and better health conditions in animals [68]. Previous research has demonstrated that n-3PUFA can enhance immunity by increasing lysozyme activity [69]. Combined with the fatty acid composition and higher expression levels of LZM, HEPC and NRAMP in the FO, SO and RO groups in our results, this indicates that higher n-3PUFA contents in FO, SO and RO could enhance the innate immunity of largemouth bass by up-regulating the levels of these defense effectors [70]. In addition, HGB and RBC are not only responsible for transporting oxygen, but are also related to better immunity in animals [13,71]. Our results show that there were higher HGB and RBC levels in the RO groups, which suggests that RO could increase the oxygen-carrying capabilities and enhance the innate immunity of largemouth bass. However, this immune regulatory mechanism of higher HGB and RBC levels mediated by RO requires further study in the future.

In general, WBC, primarily including MON, LYM and NEU, are crucial parts of cellular and humoral immune responses due to their roles in defense and immunity [72]. In this study, we found that there were lower amounts of LYM and higher amounts of WBC, NEU and MON in the SO groups, which was similar to the results obtained in myeloid-specific FoxO knockout mice [73]. Previous studies have also found that excessive amounts of WBC, NEU and MON could induce inflammatory responses in humans [74]. And excessive dietary LA contents could trigger pro-inflammatory processes by increasing the production of plasma tumor necrosis factor α in rats [75]. Meanwhile, studies have found that unnatural rises in the count of PLT often occur during in inflammatory responses [76]. Combined with these findings, this indicates that inflammatory responses might be mediated by WBC and PLT induced by higher levels of LA enriched in dietary SO in largemouth bass. Furthermore, this typical chronic inflammation is always related with weak immunity and health status in animals [13]. Inflammatory responses are mainly modulated by these so-called pro-inflammatory factors (IL-1β, IL-8, IL-12, IL-15, etc.) and anti-inflammatory molecules (TGF-β1) [77,78]. Previous studies have reported that IL-1β mRNA expression levels were increased in large yellow croaker fed with SO and in Amur sturgeon fed with oxidized fish oil [35,78]. In addition, there were higher serum IL-1 and IL-6 concentrations in weaned pigs fed with SO [79]. Similarly, higher transcription levels of IL-1β, IL-8, IL-12 and IL-15 were also shown in the liver of the SO groups in our results. However, adequate dietary n-3 highly unsaturated fatty acids (HUFAs) could significantly decrease the expression levels of Il-1β, Il-6 and Il-8 in the liver of golden pompano [80]. Combined with these findings and the higher levels of IL-1β, IL-8, IL-12 and IL-15 in this study, this indicates that higher LA contents in SO could induce potential inflammatory responses in largemouth bass [75]. Moreover, higher TGF-β1 levels were found in golden pompano (*Trachinotus ovatus*) fed with adequate dietary n-3 HUFAs [80]. And TGF-β1 could act as a positive function signal marker to suppress the inflammation in fish [13,80,81]. Combined with the fatty acid profiles and the expression variations in TGF-β1 in these lipid resources in this study, this might be due to the decrease in n-3PUFAs inhibiting the expression of anti-inflammatory factors [81]. From the above results, it is suggested that dietary RO improves health status by activating the immune system and inhibiting inflammatory factors in largemouth bass.

### 4.5. Muscle Quality

Previous studies have found that flesh quality was tightly related to antioxidant capacity mediated by dietary lipid sources [82]. It is well known that ROS are always produced during the metabolic process of various extrinsic nutrients, which could impact activities of antioxidant enzymes or functional proteins, including SOD, GST, CAT, GR, GPX, etc. [83]. Previous studies have found that the antioxidant capacities were reduced in large yellow croaker fed SO diets when compared with FO diets [82]. In our study, there were lower expression levels of GPX3 in the muscle of the SO groups when compared with FO and RO, which is similar to previous results in large yellow croaker [84], indicating that SO could reduce the antioxidant capacity in largemouth bass. Many studies have demonstrated that transcriptional variations in these antioxidant enzymes are mainly modulated by the Nrf2 signaling pathway in humans and animals [82,85,86]. Meanwhile, previous research has found that regulating the Nrf2 signaling pathway could improve muscle quality in hybrid bagrid catfish [87] and grass carp [88,89]. In our results, FO and RO were shown to significantly enhance Nrf2 mRNA levels in comparison with SO and PO, which is consistent with previous results in Jian carp [82]. In addition, research has also demonstrated that appropriate n-3 PUFA content in oils could enhance antioxidant capability and alleviate oxidative stress in humans, animals and fish species [78]. Combined with variations in n-3 PUFA content, this suggests that FO and RO could enhance antioxidative capacity and alleviate oxidative stress by up-regulating Nrf2 and its target genes’ expression levels, thus improving muscle quality in largemouth bass.

**Figure 7:**
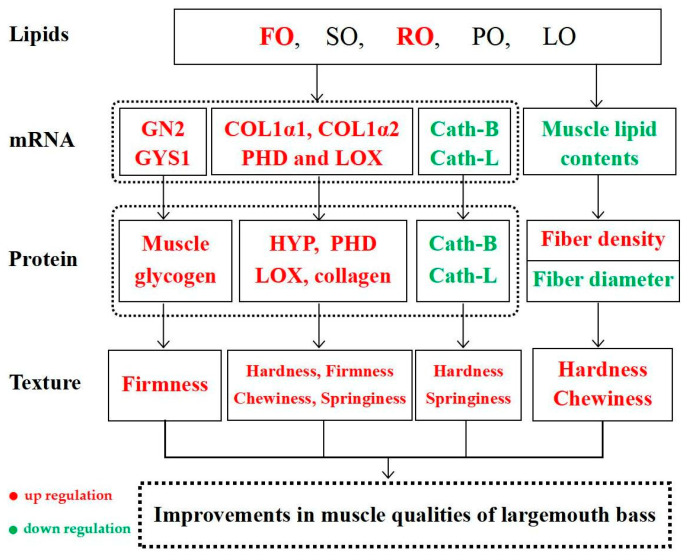
Summary of muscle quality regulated by five dietary lipid sources in largemouth bass.

Generally, texture is considered an important quality characteristic of muscle tissue. Fish muscle, unlike poultry and livestock muscle, is more easily accepted by consumers with higher muscle hardness [90]. Previous studies have also found that increasing hardness and firmness could help to improve muscle quality in large yellow croaker [91], similar to the results shown in our study. In addition, collagen content is closely correlated with hardness and firmness in fish muscle [92,93]. Meanwhile, collagen synthesis and deposition processes are mainly modulated by the activities, amounts or mRNA levels of functional enzymes or molecules, such as PHD, LOX, COL1α1, COL1α2, HYP, etc. [26,87]. Our results show that there were higher amounts of HYP, collagen, PHD and LOX, as well as higher mRNA levels of COL1α1, COL1α2 and PHD in the dorsal muscle of the FO, SO and RO trial groups, which is similar to other results in grass carp [26] and hybrid bagrid catfish [87]. Taking into consideration these previous findings and our results, this indicates that FO, SO and RO could improve muscle quality by increasing hardness, firmness and chewiness mediated by the greater collagen-synthesizing ability of large largemouth bass (Figure 7). As for lower levels of hardness, firmness and chewiness in the PO and LO groups, this may be due to the decreasing collagen contents mediated by a lower synthesizing ability [90]. In addition, previous studies have also found that there is a negative correlation between protease activity and hardness and springiness in muscle [94]. In general, lower protease activities could decelerate tissue protein hydrolysis and therefore increase muscle hardness and springiness [95]. In this study, lower levels of Cath-B and Cath-L were both present in the dorsal muscle of the FO, SO and RO groups, which is similar to previous results in hybrid bagrid catfish [87]. Combined with these findings, it is indicated that FO, SO and RO could increase muscle hardness and springiness by decreasing Cath-B and Cath-L amounts in largemouth bass (Figure 7).

In addition, hardness and chewiness are also closely related to muscle fiber diameter and density [96]. Our results found that there was a higher muscle fiber density and smaller muscle fiber diameter, as well as greater muscle hardness in the dorsal muscle of the FO, SO and RO trial groups, which is similar to previous results in glass carp [97] and Nile tilapia [96]. In addition, similar to results obtained in striped bass [98], we also found higher hardness and chewiness, higher muscle fiber density and a smaller diameter in the dorsal muscle of the FO, SO and RO groups, which indicates that FO, SO and RO could also improve muscle hardness and chewiness by modulating the muscle fiber density and diameter in largemouth bass (Figure 7) [94,98]. Meanwhile, previous studies have found that glycogen content in muscle was also related to muscle quality [99]. Glycogen synthesis could be impacted by several key genes, such as GN2 and GYS1 in animals [100]. A previous study has found that higher contents of muscle glycogen is linked with better muscle quality in Amur sturgeon [35] and higher firmness in Gilthead seabream [101]. Our results show that there were higher glycogen contents and higher mRNA levels of GN2 and GYS1 in the dorsal muscle of the FO, SO and RO groups compared with the LO groups, which is similar to previous results in Tibetan pigs [102]. Combined with these findings, it is indicated that FO, SO and RO could heighten glycogen contents by increasing mRNA levels of GN2 and GYS1, thus improving muscle quality by enhancing firmness in largemouth bass (Figure 7) [35,93].

Altered muscle fatty acid profiles could impact lipid muscle odor derived from volatile flavor compounds, including aldehyde and ketone molecules, which are mainly generated from the oxidation and decomposition of PUFAs in the muscle [21]. In this study, we found that the muscle odors were similar between FO and RO, which was in line with results obtained in large yellow croaker [21]. Previous studies have also found that n-3 PUFA-derived volatile aldehydes in fish fillets could create a pleasant odor, while n-6 PUFA-derived volatile aldehydes could exert a negative effect on muscle odor [103]. Combined with these findings, it is indicated that the similar muscle odor between the FO and RO groups was mainly mediated by similar n-3 PUFA proportions in the dorsal muscle of largemouth bass. In addition, OA can be oxidized to generate fatty, green and floral odors [104]. Meanwhile, a previous study also found that turbot fillet in FO groups could induce pleasant, green and cucumber-like odors [105]. Taking into consideration these findings in turbot and similar OA contents in RO and PO, it is indicated that there was a similar muscle odor in the FO, RO and PO groups because they could generate similar green and floral odors. However, there were significant variations in the odor of muscle between the FO and SO trial groups, may be due to the different contents of OA, LA, EPA and n-6 PUFAs in the dorsal muscle of the FO and SO trial groups [21]. Similar to previous results in large yellow croaker [21] and tench [103], n-6 PUFAs enriched in SO can derive aldehydes, which induce an unpleasant odor different to the muscle odors produced due to FO, RO and PO in largemouth bass. Previous studies have found that there are strong relationships between these volatiles and fatty acid profiles in fish muscle mediated by extrinsic lipid resources [21,103]. Combined with our results, this indicates that these distinct odor differences between LO and FO were mainly mediated by their different fatty acid profiles and lipid-derived volatiles derived from LO and FO [21,106].

## 5. Conclusions

In conclusion, FO, SO and RO could increase weight gain and n-3 PUFA content in the dorsal muscle of largemouth bass. Meanwhile, FO and RO could significantly increase the count of LYM and mRNA levels of hepatic LZM and HEPC compared with dietary PO. Meanwhile, SO could increase counts of WBC, NEU and MON, as well as mRNA levels of pro-inflammatory cytokines (IL-1β, IL-8, IL-12 and IL-15). In addition, FO and RO could improve the hardness, firmness, chewiness and springiness of muscle by increasing the collagen-synthesizing ability and decreasing the protease activity in largemouth bass. These results could provide relative references for the application of dietary lipid sources during the feeding process of juvenile and adult largemouth bass.

## Figures and Tables

**Table 1:** Composition and nutrient levels of experimental diets (air-dried basis).

Ingredient (%)	Composition of Diets (%)				
	FO	SO	RO	PO	LO
Casein ^a^	35.00	35.00	35.00	35.00	35.00
Defatted fish meal (DFM) ^b^	20.00	20.00	20.00	20.00	20.00
Gelatin ^c^	5.00	5.00	5.00	5.00	5.00
Lipid source ^d^	10.00	10.00	10.00	10.00	10.00
Dextrin ^c^	10.00	10.00	10.00	10.00	10.00
Mineral premix ^e^	2.40	2.40	2.40	2.40	2.40
Vitamin premix ^f^	1.20	1.20	1.20	1.20	1.20
Choline chloride ^g^	0.40	0.40	0.40	0.40	0.40
Microcrystalline cellulose ^h^	16.00	16.00	16.00	16.00	16.00
Proximate composition (%)					
Crude protein	50.38	50.25	50.34	50.45	50.01
Crude lipid	9.30	9.32	9.38	9.29	9.35
Ash	4.94	4.80	4.87	4.79	4.89

^a^ Supplied by Gansu Hualing Dairy Co. Ltd., (Lanzhou, China), crude protein 88.52%. ^b^ Defatted fish meal was obtained from Peruvian red fish meal supplied by Zhejiang Dongyu Biotechnology Co. Ltd. (Huzhou, China). Crude protein 75.6%, crude lipids 1.8%, fatty acid (%): C6:0, 0.14; C8:0, 0.14; C10:0, 0.14; C11:0, 0.15; C12:0, 0.27; C13:0, 0.25; C14:0, 12.30; C15:0, 0.97; C16:0, 32.13; C17:0, 1.37; C18:0, 6.09; C20:0, 0.80; C21:0, 0.53; C22:0, 0.59; C23:0, 0.53; C24:0, 0.68; SFA (saturated fatty acid), 57.07; C14:1, 0.34; C16:1, 14.48; C18:1n-9 (oleic acid, OA), 9.76; C20:1, 1.35; C24:1, 0.95; MUFA (monounsaturated fatty acid), 26.88; C18:2n-6 (linoleic acid, LA), 1.35; C18:3n-6, 0.66; C20:3n-6, 0.53; C20:4n-6 (arachidonic acid, ARA), 0.75; n-6 PUFA (n-6 polyunsaturated fatty acid), 3.28; C18:3n-3 (α-Linolenic acid, ALA), 0.78; C20:3n-3, 0.50; C20:5n-3 (eicosapentaenoic acid, EPA), 6.29; C22:6n-3 (docosahexaenoic acid, DHA), 2.48; n-3 PUFA (n-3 polyunsaturated fatty acid), 10.05; n-3 LC-PUFA (n-3 long-chain polyunsaturated fatty acid), 9.27. ^c^ Supplied by Sinopharm Chemical Reagent Co. Ltd., Shanghai, China; crude protein 87.17%. ^d^ FO was supplied by Zhejiang Dongyu Biotechnology Co. Ltd. (Huzhou, China). RO, SO and PO were pressed with an oil press from rapeseed, peanut and soybean purchased from the Milan supermarket (Huzhou, China), respectively. LO was purchased from Linyi Xincheng Jinluo Meat Products Group Co. Ltd. (Linyi, China). ^e^ Mineral mixtures (mg or g Kg^−1^ diet): NaCl, 126.09 mg; MgSO_4_·7H_2_O, 1279.2 mg; FeSO_4_·7H_2_O, 482.53 mg; ZnSO_4_·H_2_O, 441.54 mg; MnSO_4_·H_2_O, 138.27 mg; CuSO_4_·5H_2_O, 19.53 mg; CoCl_2_·6H_2_O (1%), 52.44 mg; KI, 2.61 mg; Na_2_SeO_3_, 2.63 mg; Ca(H_2_PO_4_)_2_·H_2_O, 1.5 g; microcrystalline cellulose, 2.45 g. ^f^ Vitamin premix (mg or g Kg^−1^ diet): thiamin, 20 mg; riboflavin, 50 mg; pyridoxine, 30 mg; vitamin B_12_, 0.35 mg; vitamin K_3,_ 15 mg; inositol, 600 mg; pantothenic acid, 60 mg; niacin acid, 80 mg; folic acid, 10 mg; biotin, 2 mg; vitamin A, 40 mg; vitamin D_3_, 6 mg; vitamin E, 300 mg; vitamin C, 1 g; microcrystalline cellulose, 6.29 g. ^g^ Supplied by Chemical Reagent Co., Ltd. (Shanghai, China). ^h^ Supplied by Sigma Chemical, Reagent Co., Ltd. (Shanghai, China), model 680057611.

**Table 2:** Fatty acid composition of DFM and diets (percentage of total fatty acids).

Items	Dietary Lipid Sources					
	DFM	FO	SO	RO	PO	LO
C6:0	0.14	0.13	0.12	0.46	0.11	0.12
C8:0	0.14	0.09	0.08	0.34	0.07	0.09
C10:0	0.14	0.16	0.15	0.34	0.14	0.20
C11:0	0.15	0.03	0.03	0.06	0.03	0.03
C12:0	0.27	0.17	0.16	0.35	0.14	0.21
C13:0	0.25	0.05	0.04	0.10	0.04	0.04
C14:0	12.30	7.03	1.00	2.04	0.89	2.01
C15:0	0.97	0.67	0.16	0.35	0.15	0.18
C16:0	32.13	22.39	13.72	9.19	12.34	25.21
C17:0	1.37	0.74	0.23	0.40	0.20	0.33
C18:0	6.09	5.36	4.35	2.34	5.63	14.20
C20:0	0.80	0.82	0.36	1.15	1.84	0.30
C21:0	0.53	0.12	0.11	0.22	0.10	0.10
C22:0	0.59	0.36	0.33	0.68	2.54	0.12
C23:0	0.53	0.13	0.11	0.23	0.11	0.10
C24:0	0.68	0.20	0.16	0.42	0.84	0.14
∑SFA	57.07	38.43	21.09	18.67	25.16	43.40
C14:1	0.34	0.09	0.08	0.17	0.08	0.09
C16:1	14.48	5.65	0.65	0.82	0.56	2.50
C18:1n-9 (OA)	9.76	23.23	21.89	47.51	36.22	36.34
C20:1	1.35	5.28	0.27	1.30	0.76	0.75
C24:1	0.95	1.64	0.12	0.64	0.12	0.14
∑MUFA	26.88	35.99	23.02	50.43	37.74	39.82
C18:2n-6 (LA)	1.35	8.73	45.89	23.94	35.76	14.14
C18:3n-6	0.66	0.11	0.10	0.20	0.10	0.13
C20:3n-6	0.53	0.10	0.09	0.21	0.08	0.14
C20:4n-6 (ARA)	0.75	0.79	0.10	0.19	0.09	0.20
n-6 PUFA	3.28	9.72	46.18	24.53	36.04	14.61
C18:3n-3 (ALA)	0.78	2.16	8.84	5.44	0.20	0.63
C20:3n-3	0.50	0.10	0.09	0.19	0.08	0.15
C20:5n-3 (EPA)	6.29	6.62	0.37	0.21	0.36	0.40
C22:6n-3 (DHA)	2.48	6.75	0.21	0.07	0.21	0.25
n-3 PUFA	10.05	15.63	9.50	5.91	0.85	1.43
n-3 LC-PUFA	9.27	13.47	0.66	0.47	0.65	0.80
PUFA	13.34	28.14	55.68	30.45	36.89	16.04
DHA/EPA	0.39	1.02	0.57	0.34	0.57	0.61
n-3/n-6	3.06	1.61	0.21	0.24	0.02	0.10

Note: SFA: C6:0, C8:0, C10:0, C11:0, C12:0, C13:0, C14:0, C15:0, C16:0, C17:0, C18:0, C20:0, C21:0, C22:0, C23:0 and C24:0; MUFA, monounsaturated fatty acid: C14:1, C16:1, C18:1n-9, C20:1 and C24:1; n-6 PUFA, n-6 polyunsaturated fatty acid: C18:2n-6, C18:3n-6, C20:3n-6 and C20:4n-6; n-3 PUFA, n-3 polyunsaturated fatty acid: C18:3n-3, C20:3n-3, C20:5n-3 and C22:6n-3; n-3 LC-PUFA, n-3 long-chain polyunsaturated fatty acid: C20:3n-3, C20:5n-3 and C22:6n-3; PUFA, polyunsaturated fatty acid: n-3 PUFA and n-6 PUFA.

**Table 3:** Primers for real-time qPCR used in this study.

Gene	Primers	Primer Sequence (5′–3′)	Reference	Product Length
*LZM*	F	TCATTGCTGCCATCATCTC	XM_038713810.1	115
	R	TCAACCTGCATCAGTCCC		
*HEPC*	F	GCTCTGCCGTCCCATTCA	XM_038710826.1	106
	R	CCACGATTCCATTGACATTTCTTGA		
*NRAMP*	F	TCATTCCCATCCTCACTTTC	XM_038723474.1	143
	R	TGCAGTAACATACACCACGAC		
*TGFβ1*	F	TGCGGAACTGGCTCAAAG	XM_038693206.1	111
	R	TCCCAGAAATGCCGAAAC		
*IL1β*	F	CAATGTCGCCAGACTGAA	XM_038733429.1	138
	R	GGGTGATGTGGTGGTTGA		
*IL8*	F	TTCTCCTGGCTGCTTTGG	XM_038704093.1	115
	R	TGGATGGCCCTCCTGTTA		
*IL12*	F	CCGCTGTTATTCAGTCTTACC	XM_038693841.1	117
	R	GCATCAGGGAGCAGTTCA		
*IL15*	F	TTCAGAAATCCGATGTGGC	XM_038693994.1	101
	R	GTCGATGGTGGGCGTGTA		
*Cu/Zn-SOD*	F	TAAGGCTATCTGGAATATCATCAAC	XM_038708943.1	146
	R	AATCGCCCTCCTGCTCAA		
*Mn-SOD*	F	CAGGGATCTACAGGTCTCATTC	XM_038727054.1	139
	R	GACGCTCGCTCACATTCTC		
*CAT*	F	TGCTGTCCGCTTCTCCAC	XM_038704976.1	105
	R	TCCCAGTTGCCCTCCTCA		
*GPX3*	F	CCCTCCAGTTGGAAACGA	XM_038699914.1	139
	R	ACTTGGGTGCCACCTCAT		
*GR*	F	CACGAGCAGGAAGAGTCAG	XM_038700350.1	144
	R	GCTTTGGTAGCACCCATTT		
*GST-omega*	F	GGCTTTCACCACCTATGC	XM_038739072.1	124
	R	TTCAGACTTTCTGCCCACA		
*Nrf2*	F	AAGACAAGCGTAAGAAGCG	XM_038720536.1	107
	R	CAGGCAGATTGATAATCATAGA		
*Keap1a*	F	AGGTGGTGGGAAGACTTATTG	XM_038728593.1	150
	R	GCTCCAGGTGCTTAGTGAGG		
*Keap1b*	F	TGAACGAGCTGCGTCTGG	XM_038713667.1	139
	R	TTGGTGAACATAGCCCTAAAGA		
*COL1* *α* *1*	F	TCTGGTTCGGCGAGACAATG	XM_038692282.1	108
	R	TGGACATGAGACGCAGGAAAGT		
*COL1* *α* *2*	F	TTCTGCGACTTCACCACCCG	XM_038724497.1	108
	R	TCCGAACCAGACGTGCTTTT		
*PHD*	F	GTTCTGTATTGGACGCTCTGT	XM_038711098.1	137
	R	CCGCCTTCTGCAACTTTT		
*LOX*	F	TATTTGGCACGCCGCTTTG	XM_038733746.1	115
	R	GCCGCTCTTTGGTTATCTCCTT		
*Cath-B*	F	GGCTTTGGATGTAATGGTGG	XM_038701777.1	116
	R	GGGATGGTGTAGGGACGA		
*Cath-L*	F	CAGACTGGTGCTGGTGCA	XM_038729783.1	113
	R	GGGAAATCAGGCGTTTGTAC		
*GYS1*	F	AAGACGAACGCTATGACGAG	XM_038697432.1	140
	R	TTTCACGCTTGCGACACC		
*GN2*	F	CACGCAGCATTGTTGTCA	XM_038722837.1	126
	R	AGGCCAGATGTAGAGGGTC		
*β-action*	F	GCGTGACATCAAGGAGAAGC	XM_038695351.1	149
	R	CTGGGCAACGGAACCTCT		

F: forward; R: reverse; LZM, lysozyme; HEPC, hepcidin; NRAMP, natural resistance-associated macrophage protein; TGF-β1, transforming growth factor beta 1; IL-1β, interleukin-1β; IL-8, interleukin 8; IL-12, interleukin 12; IL-15, interleukin 15; Cu/Zn-SOD, Cu/Zn-Superoxide dismutase; Mn-SOD, Mn-Superoxide dismutase; CAT, catalase; GPX3, glutathione peroxidase 3; GR, glutathione reductase; GST-omega, glutathione S-transferase omega; Nrf2, NF-E2-related factor 2; Keap1a, kelch-like ECH-associated protein 1a; Keap1b, kelch-like ECH-associated protein 1b; collagen 1α1, COL1α1; collagen 1α2, COL1α2; PHD, prolyl hydroxylase; Cath-B, cathepsin-b; Cath-L, cathepsin-l; LOX, lysyloxidase; GYS1, glycogen synthase 1; GN2, glycogenin 2.

**Table 4:** Effects of different dietary lipid sources on growth performance of largemouth bass.

Items	Dietary Lipid Sources							
	FO	SO	RO	PO	LO	AN	LT	QT
IBW (g)	9.33 ± 0.01	9.33 ± 0.01	9.34 ± 0.01	9.32 ± 0.01	9.32 ± 0.01	0.149	-	-
FBW (g)	30.85 ± 0.45 ^c^	29.93 ± 0.58 ^c^	30.85 ± 0.93 ^c^	27.21 ± 0.37 ^b^	25.31 ± 0.20 ^a^	0.000	0.000	0.006
WG (%)	230.46 ± 5.07 ^c^	220.91 ± 6.00 ^c^	230.49 ± 9.64 ^c^	191.81 ± 3.77 ^b^	171.57 ± 2.42 ^a^	0.000	0.000	0.006
SGR (%/d)	2.44 ± 0.03 ^c^	2.38 ± 0.04 ^c^	2.44 ± 0.06 ^c^	2.19 ± 0.03 ^b^	2.04 ± 0.02 ^a^	0.000	0.000	0.003
FCR	1.03 ± 0.04 ^a^	1.08 ± 0.05 ^a^	1.06 ± 0.05 ^a^	1.15 ± 0.08 ^a^	1.47 ± 0.03 ^b^	0.000	0.000	0.000
PER (%)	1.92 ± 0.06 ^b^	1.85 ± 0.08 ^b^	1.88 ± 0.08 ^b^	1.72 ± 0.11 ^b^	1.36 ± 0.03 ^a^	0.000	0.000	0.001
HSI (%)	1.16 ± 0.04 ^a^	1.28 ± 0.08 ^ab^	1.35 ± 0.07 ^b^	1.16 ± 0.05 ^a^	1.23 ± 0.03 ^ab^	0.009	0.952	0.062
VSI (%)	8.55 ± 0.34 ^bc^	8.16 ± 0.04 ^ab^	8.71 ± 0.12 ^c^	8.33 ± 0.08 ^abc^	7.90 ± 0.18 ^a^	0.002	0.061	0.120
IPF (%)	2.33 ± 0.08 ^ab^	2.53 ± 0.08 ^b^	2.11 ± 0.07 ^a^	2.27 ± 0.17 ^ab^	2.43 ± 0.04 ^b^	0.004	0.886	0.196
CF (g/cm^3^)	2.15 ± 0.02 ^b^	2.06 ± 0.03 ^ab^	2.05 ± 0.05 ^ab^	1.96 ± 0.06 ^a^	2.15 ± 0.01 ^b^	0.001	0.560	0.002
CR (%)	40.79 ± 0.29 ^ab^	41.47 ± 1.42 ^b^	42.17 ± 1.68 ^b^	38.97 ± 1.31 ^ab^	37.60 ± 0.93 ^a^	0.006	0.010	0.014
SR (%)	98.89 ± 1.92	97.78 ± 1.92	100.00 ± 0.00	96.67 ± 3.34	94.44 ± 1.93	0.068	-	-

Note: IBW, initial body weight; FBW, final body weight; WG, weight gain; SGR, special growth rate; FCR, feed conversion ratio; PER, protein efficiency ratio; HSI, hepatosomatic index; VSI, viscerosomatic index; IPF, intraperitoneal fat body index; CF, condition factor; CR, carcass ratio, SR, survival ratio. AN, ANOVA; LT, linear trend; QT, quadratic trend. The following table is the same. Values are means ± SD (*n* = 15). Mean values with different superscripts in the same row are significantly different based on Tukey’s test (*p* < 0.05).

**Table 5:** Effects of different dietary lipid sources on whole-body and dorsal muscle nutritional composition of largemouth bass.

Body Composition (%)	Dietary Lipid Sources							
	FO	SO	RO	PO	LO	AN	LT	QT
Whole body								
Moisture	71.45 ± 0.20 ^c^	70.49 ± 0.05 ^a^	72.07 ± 0.17 ^d^	71.05 ± 0.12 ^b^	72.00 ± 0.13 ^d^	0.000	0.153	0.367
Crude protein	17.35 ± 0.37	17.45 ± 0.21	17.21 ± 0.18	17.02 ± 0.26	17.09 ± 0.16	0.262	-	-
Crude lipid	7.43 ± 0.20 ^c^	8.15 ± 0.09 ^d^	6.87 ± 0.16 ^ab^	6.82 ± 0.13 ^a^	7.23 ± 0.12 ^bc^	0.000	0.062	0.563
Ash	3.60 ± 0.16	3.73 ± 0.07	3.58 ± 0.15	3.88 ± 0.15	3.60 ± 0.16	0.116	-	-
Dorsal muscle								
Moisture	75.12 ± 0.47 ^b^	74.64 ± 0.48 ^b^	75.11 ± 0.58 ^b^	74.67 ± 0.22 ^b^	73.37 ± 0.48 ^a^	0.005	0.007	0.042
Crude protein	21.98 ± 0.23	21.80 ± 0.43	21.19 ± 0.29	21.51 ± 0.32	21.81 ± 0.34	0.093	-	-
Crude lipid	1.19 ± 0.10 ^a^	1.31 ± 0.09 ^a^	1.23 ± 0.10 ^a^	1.29 ± 0.11 ^a^	1.70 ± 0.09 ^b^	0.000	0.003	0.022
Ash	1.04 ± 0.07	1.02 ± 0.10	1.03 ± 0.04	1.04 ± 0.04	1.07 ± 0.04	0.869	-	-

Values are means ± SD (*n* = 9). Mean values with different superscripts in the same row are significantly different based on Tukey’s test (*p* < 0.05).

**Table 6:** Effects of different dietary lipid sources on fatty acid composition of dorsal muscle of largemouth bass (percentage of total fatty acids).

Items	Dietary Lipid Sources							
	FO	SO	RO	PO	LO	AN	LT	QT
C10:0	0.40 ± 0.05 ^b^	0.26 ± 0.03 ^a^	0.74 ± 0.02 ^c^	0.31 ± 0.01 ^a^	0.34 ± 0.04 ^ab^	0.000	0.838	0.369
C11:0	-	0.25 ± 0.02 ^ab^	0.84 ± 0.03 ^d^	0.27 ± 0.01 ^b^	0.23 ± 0.02 ^a^	0.000	0.390	0.001
C12:0	0.51 ± 0.05 ^b^	0.34 ± 0.04 ^a^	0.98 ± 0.01 ^c^	0.48 ± 0.04 ^b^	0.51 ± 0.04 ^b^	0.000	0.851	0.115
C13:0	0.60 ± 0.07 ^b^	0.39 ± 0.01 ^a^	1.05 ± 0.05 ^c^	0.55 ± 0.02 ^b^	0.52 ± 0.03 ^b^	0.000	0.994	0.133
C14:0	1.33 ± 0.07 ^ab^	1.19 ± 0.03 ^a^	1.91 ± 0.02 ^c^	1.21 ± 0.11 ^a^	1.52 ± 0.08 ^b^	0.000	0.471	0.422
C15:0	0.89 ± 0.04 ^b^	0.69 ± 0.02 ^a^	1.81 ± 0.06 ^c^	0.95 ± 0.04 ^b^	0.88 ± 0.03 ^b^	0.000	0.762	0.057
C16:0	14.08 ± 1.65 ^a^	17.07 ± 0.03 ^b^	12.21 ± 0.02 ^a^	17.11 ± 0.08 ^b^	21.03 ± 0.09 ^c^	0.000	0.926	0.114
C17:0	1.36 ± 0.16 ^b^	0.92 ± 0.03 ^a^	2.36 ± 0.07 ^c^	1.24 ± 0.06 ^b^	1.15 ± 0.03 ^b^	0.000	0.048	0.003
C18:0	6.01 ± 0.28 ^bc^	5.64 ± 0.03 ^ab^	5.29 ± 0.05 ^a^	6.35 ± 0.10 ^c d^	6.49 ± 0.08 ^d^	0.000	0.737	0.055
C20:0	1.42 ± 0.04 ^b^	1.05 ± 0.02 ^a^	2.90 ± 0.05 ^d^	1.55 ± 0.05 ^c^	1.38 ± 0.04 ^b^	0.000	0.829	0.043
C21:0	-	-	-	0.33 ± 0.02 ^b^	0.16 ± 0.01 ^a^	0.000	0.004	0.955
C22:0	1.56 ± 0.09 ^d^	0.93 ± 0.12 ^b^	0.49 ± 0.02 ^a^	1.37 ± 0.04 ^cd^	1.26 ± 0.07 ^c^	0.000	0.838	0.003
C23:0	-	0.98 ± 0.05 ^b^	-	0.34 ± 0.01 ^a^	-	0.000	0.394	0.130
C24:0	2.16 ± 0.02 ^d^	1.25 ± 0.05 ^b^	0.64 ± 0.03 ^a^	1.79 ± 0.05 ^c^	0.75 ± 0.07 ^a^	0.000	0.034	0.204
∑SFA	30.32 ± 1.00 ^a^	30.95 ± 0.21 ^a^	31.20 ± 0.09 ^a^	33.51 ± 0.12 ^b^	36.06 ± 0.18 ^c^	0.000	0.000	0.000
C14:1	0.32 ± 0.02 ^a^	0.59 ± 0.02 ^c^	1.15 ± 0.03 ^d^	0.46 ± 0.04 ^b^	0.39 ± 0.02 ^a^	0.000	1.000	0.001
C16:1	1.71 ± 0.11 ^a^	1.68 ± 0.03 ^a^	2.64 ± 0.07 ^b^	1.76 ± 0.02 ^a^	3.06 ± 0.03 ^c^	0.000	0.050	0.414
C18:1n-9	17.08 ± 0.15 ^a^	18.57 ± 1.04 ^b^	24.89 ± 0.08 ^d^	24.27 ± 0.14 ^c d^	23.24 ± 0.09 ^c^	0.000	0.000	0.002
C20:1	2.29 ± 0.15 ^b^	1.62 ± 0.07 ^a^	3.61 ± 0.06 ^c^	2.49 ± 0.07 ^b^	2.45 ± 0.08 ^b^	0.000	0.344	0.291
C24:1	-	1.31 ± 0.05 ^b^	-	0.46 ± 0.02 ^a^	-	0.000	0.399	0.218
∑MUFA	21.40 ± 0.30 ^a^	23.76 ± 0.97 ^b^	32.30 ± 0.10 ^d^	29.44 ± 0.09 ^c^	29.13 ± 0.01 ^c^	0.000	0.001	0.002
C18:2n-6	22.13 ± 0.62 ^e^	26.50 ± 0.04 ^d^	9.69 ± 0.01 ^a^	19.31 ± 0.13 ^c^	17.42 ± 0.15 ^b^	0.000	0.118	0.251
C18:3n-6	1.69 ± 0.12 ^c^	1.20 ± 0.02 ^a^	2.90 ± 0.08 ^d^	1.63 ± 0.10 ^bc^	1.42 ± 0.06 ^ab^	0.000	0.924	0.080
C20:3n-6	2.32 ± 0.14 ^b^	1.72 ± 0.09 ^a^	2.85 ± 0.12 ^c^	2.21 ± 0.06 ^b^	1.80 ± 0.03 ^a^	0.000	0.501	0.143
C20:4n-6	2.28 ± 0.12 ^b^	1.64 ± 0.19 ^a^	2.77 ± 0.05 ^c^	2.38 ± 0.05 ^b^	1.86 ± 0.07 ^a^	0.000	0.912	0.183
n-6PUFA	28.42 ± 0.96 ^d^	31.06 ± 0.26 ^e^	18.22 ± 0.13 ^a^	25.53 ± 0.07 ^c^	22.49 ± 0.26 ^b^	0.000	0.035	0.335
C18:3n-3	2.35 ± 0.06 ^b^	2.31 ± 0.03 ^b^	3.73 ± 0.05 ^c^	1.53 ± 0.06 ^a^	1.51 ± 0.05 ^a^	0.000	0.111	0.027
C20:3n-3	1.46 ± 0.12 ^b^	1.00 ± 0.11 ^a^	2.68 ± 0.08 ^c^	-	1.27 ± 0.04 ^b^	0.000	0.416	0.661
C20:5n-3	3.37 ± 0.01 ^e^	1.21 ± 0.19 ^a^	3.11 ± 0.07 ^c^	1.56 ± 0.05 ^b^	1.61 ± 0.06 ^b^	0.000	0.055	0.060
C22:6n-3	8.66 ± 0.14 ^c^	5.88 ± 0.05 ^b^	5.12 ± 0.04 ^a^	5.30 ± 0.09 ^a^	5.19 ± 0.09 ^a^	0.000	0.000	0.000
n-3PUFA	15.83 ± 0.04 ^e^	10.39 ± 0.33 ^c^	14.64 ± 0.14 ^d^	8.38 ± 0.10 ^a^	9.57 ± 0.07 ^b^	0.000	0.004	0.591
n-3LC-PUFA	13.49 ± 0.09 ^d^	8.08 ± 0.30 ^b^	10.91 ± 0.09 ^c^	6.85 ± 0.05 ^a^	8.06 ± 0.02 ^b^	0.000	0.003	0.110
PUFA	44.25 ± 0.96 ^d^	41.45 ± 0.55 ^c^	32.86 ± 0.14 ^ab^	33.92 ± 0.17 ^b^	32.07 ± 0.25 ^a^	0.000	0.000	0.012
DHA/EPA	2.56 ± 0.04 ^ab^	4.96 ± 0.86 ^c^	1.65 ± 0.03 ^a^	3.41 ± 0.16 ^b^	3.22 ± 0.17 ^b^	0.000	0.992	0.974
n-3/n-6	0.56 ± 0.02 ^c^	0.33 ± 0.01 ^a^	0.80 ± 0.01 ^d^	0.33 ± 0.00 ^a^	0.43 ± 0.01 ^b^	0.000	0.442	0.483

Note: “-”, not detected. Some fatty acids, of which the contents are minor, trace amount or not detected, such as 20:2n-6, 22:4n-6, 18:4n-3, 22:3n-3 and 22:5n-3, were not listed in the table. Values are means ± SD (*n* = 9). Mean values with different superscripts in the same row are significantly different based on Tukey’s test (*p* < 0.05).

**Table 7:** Effects of different dietary lipid sources on hematological parameters in largemouth bass.

Items	Dietary Lipid Sources							
	FO	SO	RO	PO	LO	AN	LT	QT
WBC (10^9^/L)	165.57 ± 7.92 ^a^	191.04 ± 8.06 ^b^	166.27 ± 6.81 ^a^	160.81 ± 5.94 ^a^	156.23 ± 2.15 ^a^	0.001	0.045	0.120
RBC (10^12^/L)	2.39 ± 0.09 ^a^	2.77 ± 0.12 ^bc^	2.84 ± 0.19 ^c^	2.47 ± 0.10 ^ab^	2.43 ± 0.10 ^a^	0.003	0.593	0.003
HGB (g/L)	70.67 ± 5.51 ^a^	80.67 ± 7.02 ^ab^	86.33 ± 7.37 ^b^	71.33 ± 2.08 ^a^	73.67 ± 1.53 ^ab^	0.019	0.822	0.030
PLT (10^9^/L)	84.50 ± 1.00 ^a^	124.33 ± 11.24 ^c^	90.00 ± 2.65 ^a^	119.00 ± 8.05 ^bc^	99.75 ± 8.47 ^ab^	0.000	0.449	0.158
MCH (pg)	28.40 ± 0.46 ^a^	29.40 ± 0.79 ^abc^	28.60 ± 0.66 ^ab^	30.13 ± 0.25 ^bc^	30.20 ± 0.56 ^c^	0.008	0.004	0.766
MCV (fL)	132.47 ± 1.30 ^a^	131.47 ± 1.36 ^a^	133.03 ± 1.75 ^a^	131.48 ± 4.79 ^a^	146.80 ± 3.21 ^b^	0.000	0.010	0.003
MCHC (g/L)	210.67 ± 3.79 ^ab^	255.33 ± 3.06 ^c^	214.33 ± 3.06 ^ab^	226.17 ± 14.34 ^b^	201.33 ± 3.51 ^a^	0.000	0.206	0.037
NEU (10^9^/L)	12.94 ± 1.72 ^b^	34.77 ± 2.73 ^c^	11.80 ± 1.01 ^b^	4.83 ± 0.94 ^a^	7.33 ± 0.54 ^a^	0.000	0.036	0.296
LYM (10^9^/L)	140.96 ± 3.69 ^b^	120.51 ± 4.39 ^a^	142.62 ± 2.24 ^b^	139.92 ± 3.23 ^b^	136.37 ± 1.54 ^b^	0.000	0.541	0.660
MON (10^9^/L)	10.47 ± 2.00 ^a^	27.85 ± 1.48 ^c^	16.04 ± 1.78 ^b^	13.88 ± 0.68 ^ab^	12.40 ± 0.88 ^ab^	0.000	0.412	0.041

Note: WBC, white blood cells; RBC, red blood cells; HGB, hemoglobin; PLT, platelets; MCH, mean corpuscular Hb; MCV, mean corpuscular volume; MCHC, mean corpuscular Hb concentration; NEU, neutrophils; LYM, lymphocytes; MON, monocytes. Values are means ± SD (*n* = 15). Mean values with different superscripts in the same row are significantly different based on Tukey’s test (*p* < 0.05).

**Table 8:** Effects of different dietary lipid sources on serum biochemical parameters in largemouth bass.

Items	Dietary Lipid Sources							
	FO	SO	RO	PO	LO	AN	LT	QT
HDL (mmol/L)	2.21 ± 0.40	2.29 ± 0.19	2.52 ± 0.38	2.11 ± 0.04	2.80 ± 0.09	0.054	-	-
LDL (mmol/L)	2.01 ± 0.10 ^a^	2.06 ± 0.04a ^b^	2.38 ± 0.11 ^bc^	2.01 ± 0.18 ^a^	2.61 ± 0.16 ^c^	0.000	0.014	0.406
GLU (mmol/L)	8.35 ± 0.96 ^a^	10.91 ± 0.45 ^b^	12.41 ± 0.27 ^c^	8.12 ± 0.23 ^a^	10.30 ± 0.40 ^b^	0.000	0.739	0.089
TG (mmol/L)	4.14 ± 0.69 ^a^	6.69 ± 0.36 ^b^	4.83 ± 0.51 ^a^	4.35 ± 0.23 ^a^	6.23 ± 0.47 ^b^	0.000	0.396	0.992
TC (mmol/L)	5.32 ± 0.16 ^a^	5.79 ± 0.11 ^b^	6.34 ± 0.09 ^c^	5.72 ± 0.20 ^b^	6.83 ± 0.09 ^d^	0.000	0.001	0.899
BUN (mmol/L)	2.45 ± 0.28 ^ab^	2.37 ± 0.19 ^ab^	2.34 ± 0.31 ^a^	2.70 ± 0.21 ^bc^	2.94 ± 0.10 ^c^	0.003	0.017	0.009
ALB (g/L)	6.85 ± 0.10 ^b^	6.21 ± 0.10 ^a^	6.38 ± 0.16 ^a^	6.57 ± 0.06 ^ab^	7.30 ± 0.26 ^c^	0.000	0.341	0.812
ALP (U/L)	70.80 ± 0.46 ^c^	53.03 ± 1.34 ^a^	81.83 ± 0.61 ^d^	61.43 ± 6.10 ^ab^	61.72 ± 3.53 ^b^	0.000	0.099	0.000
AST (U/L)	14.43 ± 1.28 ^a^	24.03 ± 0.15 ^c^	13.21 ± 0.92 ^a^	20.64 ± 0.51 ^b^	19.96 ± 0.41 ^b^	0.000	0.629	0.596
ALT (U/L)	2.25 ± 0.25 ^b^	2.18 ± 0.13 ^b^	1.58 ± 0.03 ^a^	2.08 ± 0.16 ^b^	2.50 ± 0.17 ^b^	0.001	0.548	0.002

Note: HDL-C, high-density lipoprotein cholesterol; LDL-C, low-density lipoprotein cholesterol; GLU, glucose; TG, triglyceride; TC, total cholesterol; BUN, blood urea nitrogen; ALB, albumin; ALP, alkaline phosphatase; AST, aspartate aminotransferase; ALT, alanine aminotransferase. Values are means ± SD (*n* = 9). Mean values with different superscripts in the same row are significantly different based on Tukey’s test (*p* < 0.05).

**Table 9:** Effects of different dietary lipid sources on serum hormones and adipokines in largemouth bass.

Items	Dietary Lipid Sources							
	FO	SO	RO	PO	LO	AN	LT	QT
INS/(mIU/L)	120.95 ± 2.91	120.88 ± 0.74	118.88 ± 1.84	117.50 ± 2.15	119.86 ± 1.05	0.214	-	-
GC (ng/L)	347.73 ± 1.91 ^a^	374.46 ± 13.56 ^b^	375.55 ± 6.80 ^b^	351.38 ± 7.56 ^ab^	372.21 ± 11.96 ^ab^	0.008	0.357	0.264
APLN (mg/L)	1116.55 ± 33.16 ^b^	1035.43 ± 14.76 ^b^	1232.33 ± 55.87 ^c^	922.42 ± 37.28 ^a^	832.69 ± 19.85 ^a^	0.000	0.006	0.027
ADPN (ng/L)	19.20 ± 0.90	18.01 ± 0.85	18.62 ± 0.43	17.43 ± 1.11	18.92 ± 1.32	0.240	-	-
AGRP (mg/L)	131.04 ± 5.38	131.04 ± 1.79	134.93 ± 1.89	130.44 ± 7.23	138.46 ± 6.12	0.295	-	-

Note: INS, insulin; GC, glucagon; APLN, apelin; ADPN, adiponectin; AGRP, agouti-related proteins. Values are means ± SD (*n* = 9). Mean values with different superscripts in the same row are significantly different based on Tukey’s test (*p* < 0.05).

**Table 10:** Effects of different dietary lipid sources on the digestive enzymes in largemouth bass.

Items	Dietary Lipid Sources							
	FO	SO	RO	PO	LO	AN	LT	QT
Liver								
AMS (U/mg prot)	0.24 ± 0.01 ^c^	0.24 ± 0.01 ^c^	0.21 ± 0.01 ^c^	0.18 ± 0.01 ^b^	0.14 ± 0.01 ^a^	0.000	0.000	0.001
TRY (U/mg prot)	205.82 ± 11.08 ^bc^	212.25 ± 7.00 ^c^	209.66 ± 5.38 ^c^	186.36 ± 8.95 ^ab^	173.82 ± 8.21 ^a^	0.012	0.001	0.008
LPS (U/mg prot)	21.35 ± 2.19 ^b^	20.40 ± 1.36 ^ab^	18.73 ± 1.83 ^ab^	17.60 ± 2.97 ^ab^	15.80 ± 0.99 ^a^	0.043	0.001	0.701
Intestines								
AMS (U/mg prot)	1.44 ± 0.12 ^a^	2.02 ± 0.08 ^b^	1.60 ± 0.19 ^a^	1.71 ± 0.12 ^ab^	1.58 ± 0.04 ^a^	0.001	0.939	0.072
TRY (U/mg prot)	1621.45 ± 31.45 ^bc^	1635.07 ± 33.81 ^c^	2023.67 ± 19.41 ^d^	1322.17 ± 51.77 ^a^	1409.68 ± 46.55 ^ab^	0.000	0.114	0.063
LPS (U/mg prot)	41.28 ± 1.65 ^bc^	46.24 ± 4.37 ^c^	41.75 ± 1.03 ^bc^	39.37 ± 2.66 ^b^	29.79 ± 0.73 ^a^	0.487	-	-

Note: AMS, α-amylase; TRY, trypsin; LPS, lipase. Values are means ± SD (*n* = 9). Mean values with different superscripts in the same row are significantly different based on Tukey’s test (*p* < 0.05).

**Table 11:** Effects of different dietary lipid sources on muscle texture and muscle cellularity in the dorsal muscle of largemouth bass.

Items	Dietary Lipid Source							
	FO	SO	RO	PO	LO	AN	LT	QT
Hardness/(g/s)	85.16 ± 1.15 ^b^	83.50 ± 0.78 ^b^	81.69 ± 1.48 ^ab^	79.56 ± 1.80 ^a^	78.99 ± 1.77 ^a^	0.002	0.000	0.537
Toughness/mm	1.44 ± 0.03	1.44 ± 0.02	1.43 ± 0.03	1.42 ± 0.03	1.36 ± 0.07	0.098	-	-
Firmness/g	150.72 ± 3.70 ^c^	150.46 ± 1.02 ^c^	146.13 ± 5.99 ^bc^	136.87 ± 3.37 ^ab^	129.97 ± 5.24 ^a^	0.000	0.000	0.058
Chewiness/(g·s)	81.32 ± 1.45 ^b^	80.78 ± 2.85 ^b^	80.33 ± 1.83 ^ab^	75.26 ± 1.36 ^a^	75.16 ± 2.09 ^a^	0.005	0.000	0.435
Springiness/%	34.45 ± 1.19 ^b^	35.59 ± 0.81 ^b^	33.76 ± 1.53 ^b^	33.42 ± 0.87 ^b^	28.53 ± 0.48 ^a^	0.000	0.001	0.001
Myofiber density (fibers/mm^2^)	189.51 ± 12.22 ^b^	187.46 ± 5.00 ^b^	184.42 ± 3.01 ^ab^	169.53 ± 12.58 ^ab^	163.01 ± 4.22 ^a^	0.011	0.000	0.258
Myofiber diameter (µm)	45.86 ± 3.39 ^a^	48.07 ± 1.36 ^ab^	50.45 ± 0.99 ^ab^	50.74 ± 2.77 ^ab^	53.07 ± 1.00 ^b^	0.018	0.000	0.682

Values are means ± SD (*n* = 9). Mean values with different superscripts in the same row are significantly different based on Tukey’s test (*p* < 0.05).

**Table 12:** Effects of different dietary lipid sources on collagen synthesis-related indexes and cathepsin content in the dorsal muscle of largemouth bass.

Items	Dietary Lipid Source							
	FO	SO	RO	PO	LO	AN	LT	QT
HYP (μg/mg)	0.34 ± 0.01 ^c^	0.34 ± 0.01 ^c^	0.32 ± 0.01 ^bc^	0.28 ± 0.02 ^a^	0.30 ± 0.01 ^ab^	0.000	0.000	0.662
Collagen (μg/mg)	2.79 ± 0.15 ^b^	2.71 ± 0.07 ^b^	2.56 ± 0.08 ^ab^	2.39 ± 0.14 ^a^	2.38 ± 0.04 ^a^	0.002	0.000	0.667
PHD (pg/mL)	63.00 ± 4.19 ^b^	55.96 ± 6.19 ^ab^	52.43 ± 4.35 ^ab^	50.41 ± 2.05 ^a^	48.65 ± 5.25 ^a^	0.024	0.001	0.213
LOX (pg/mL)	8.24 ± 0.35 ^c^	5.79 ± 0.54 ^ab^	7.25 ± 1.05 ^bc^	4.37 ± 0.24 ^a^	6.13 ± 1.18 ^ab^	0.001	0.035	0.156
PYD (nmol/L)	46.93 ± 2.43 ^b^	44.82 ± 1.57 ^ab^	46.62 ± 2.47 ^ab^	41.30 ± 1.09 ^a^	43.86 ± 2.22 ^ab^	0.039	0.047	0.689
Cath-B (ng/mL)	302.76 ± 11.75 ^a^	303.01 ± 12.56 ^a^	321.35 ± 5.09 ^bc^	331.60 ± 11.27 ^bc^	352.11 ± 6.93 ^c^	0.001	0.000	0.142
Cath-L (ng/mL)	153.70 ± 1.77	154.86 ± 2.22	150.59 ± 5.24	157.10 ± 5.48	160.97 ± 8.79	0.253	-	-

Note: HYP, hydroxyproline; PHD, prolyl hydroxylase; LOX, lysyl oxidase; Cath-B, cathepsin-B; Cath-L, cathepsin-L; PYD, pyridine cross-linking. Values are means ± SD (*n* = 9). Mean values with different superscripts in the same row are significantly different based on Tukey’s test (*p* < 0.05).

## Data Availability

The data presented in this study are available in the main article.
